# Genome-wide SNP discovery and population structure analysis in pepper (*Capsicum annuum*) using genotyping by sequencing

**DOI:** 10.1186/s12864-016-3297-7

**Published:** 2016-11-21

**Authors:** F. Taranto, N. D’Agostino, B. Greco, T. Cardi, P. Tripodi

**Affiliations:** Consiglio per la ricerca in agricoltura e l’analisi dell’economia agraria—Centro di ricerca per l’orticoltura (CREA-ORT), Via dei Cavalleggeri 25, 84098 Pontecagnano Faiano, SA Italy

**Keywords:** *Genotyping by sequencing*, *Pepper*, *Population structure*, *Single nucleotide polymorphism*

## Abstract

**Background:**

Knowledge on population structure and genetic diversity in vegetable crops is essential for association mapping studies and genomic selection. Genotyping by sequencing (GBS) represents an innovative method for large scale SNP detection and genotyping of genetic resources. Herein we used the GBS approach for the genome-wide identification of SNPs in a collection of *Capsicum* spp. accessions and for the assessment of the level of genetic diversity in a subset of 222 cultivated pepper (*Capsicum annum*) genotypes.

**Results:**

GBS analysis generated a total of 7,568,894 master tags, of which 43.4% uniquely aligned to the reference genome CM334. A total of 108,591 SNP markers were identified, of which 105,184 were in *C. annuum* accessions. In order to explore the genetic diversity of *C. annuum* and to select a minimal core set representing most of the total genetic variation with minimum redundancy, a subset of 222 *C. annuum* accessions were analysed using 32,950 high quality SNPs. Based on Bayesian and Hierarchical clustering it was possible to divide the collection into three clusters. Cluster I had the majority of varieties and landraces mainly from Southern and Northern Italy, and from Eastern Europe, whereas clusters II and III comprised accessions of different geographical origins. Considering the genome-wide genetic variation among the accessions included in cluster I, a second round of Bayesian (K = 3) and Hierarchical (K = 2) clustering was performed. These analysis showed that genotypes were grouped not only based on geographical origin, but also on fruit-related features.

**Conclusions:**

GBS data has proven useful to assess the genetic diversity in a collection of *C. annuum* accessions. The high number of SNP markers, uniformly distributed on the 12 chromosomes, allowed the accessions to be distinguished according to geographical origin and fruit-related features. SNP markers and information on population structure developed in this study will undoubtedly support genome-wide association mapping studies and marker-assisted selection programs.

**Electronic supplementary material:**

The online version of this article (doi:10.1186/s12864-016-3297-7) contains supplementary material, which is available to authorized users.

## Background

The characterization and use of the worldwide genetic diversity is essential for food security and nutrition of future generations. Groundbreaking discoveries in molecular biology allow the identification of complex genetic networks that have further unlocked our understanding of the genetic potential of plant germplasm. This represents a key point for the progress of the genetic improvement of crops. Pepper (*Capsicum* spp.) is an economically important vegetable crop belonging to the *Solanaceae* family. The *Capsicum* genus originates from the tropical and sub-tropical regions of America. Within the *Capsicum* genus, there are at least 31 species, five of which were domesticated through distinct events at different primary diversification centres: *C. annuum*, *C. baccatum*, *C. chinense*, *C. frutescens* and *C. pubescens* [1]. Among the domesticated *Capsicum* spp*.*, *C. annuum* is the most widely grown species in the world as sweet and hot pepper [2] and it is the most used in breeding programs. *C. annuum* was domesticated in highland Mexico and comprises most of the Mexican chili peppers, most of the hot peppers from Africa and Asia and various sweet pepper cultivars growing in European temperate regions [3].

Domestication and subsequent steps of artificial selection led to the great variation in fruit size, shape, colour and pungency of contemporary *C. annuum*, depending on consumer preference and product differentiation according to regional origin [4]. Furthermore, in the last century, breeding programs resulted in the selection of commercial varieties and hybrids frequently carrying genes for resistance to diseases and pests, and higher and uniform yield [5]. As a consequence, modern cultivars have replaced the diversified and heterogeneous landraces all around the world, leading to a reduction of genetic diversity [6]. In the last decades, thanks to international agricultural policies and actions focused on the protection of biodiversity and the promotion of the sustainable use of crop resources, curated collections were constituted using landraces selected on the basis of a recognizable morphology and of adaptation to local pedo-climatic conditions [7]. The availability of large germplasm collections facilitates the evaluation of population diversity and genetic structure, providing vital information for genome-wide association mapping and allele mining studies that can be exploited by plant breeders for the development of novel varieties and seed conservation programs [8–10].

Population structure and level of genetic diversity of *Capsicum* spp. have been estimated by different approaches, including the use of biochemical, morphological and molecular markers [11–15]. Molecular markers permit in-depth characterization of germplasm [16] and improve the efficiency and precision of conventional plant breeding schemes through marked-assisted selection. Amplified Fragment Length Polymorphism (AFLP), Simple Sequence Repeat (SSR) and Single Nucleotide Polymorphism (SNP) markers proved useful in detecting genetic diversity and determining genetic relationships in pepper germplasm [13–15]. However, although AFLP and SSR markers were widely used due to their highly polymorphic nature [14, 15], this was partly related to a high genotyping error rate limiting their application for genetic studies. Indeed, AFLP and SSR gel-based genotyping is very laborious and can be affected by human errors. For those reasons, it is hard to combine and integrate information from low-throughput semi-automated fragment analysis systems. SNPs, abundant in plants, can be considered the primary choice for many genetic studies, having a number of advantages such as flexibility, reduced error rate, speed- and cost-effectiveness [17]. Recently, the availability of a variety of almost fully automated high-throughput SNP genotyping platforms has dramatically reduced costs and time associated with the development of plant breeding schemes. Most importantly, SNP markers can be easily converted to universal genotype information from different technology sources, making the integration from different SNP platforms truly effective. The advent of Next Generation Sequencing (NGS) technologies and the availability of a reference genome sequence for many crops allowed the implementation of several methods for SNPs discovery, with the Genotype by sequencing (GBS) the most recent developed [17], simultaneously allowing SNP discovery and genotyping. It does not require any *a priori* knowledge on the genome of the species of interest [17–19] and provides a rapid, high-throughput and cost-effective tool for exploring plant genetic diversity on a genome-wide scale [20–24]. In the last few years, it has been widely used to investigate genetic diversity in many crop species, such as sorghum, brassica and cotton [18], watermelon [16] and rice [25], using germplasm collections [16], recombinant inbred lines (RILs) or backcross (BC) populations [18] as starting material. To the best of our knowledge, no genetic diversity studies using GBS are available of *Capsicum* although a recent paper reports the use of GBS to confirm the genetic background of ten pepper plants deriving from a marker-assisted backcrossing scheme [26]. Although several SNP-based marker studies have been performed so far [27–30], no GBS work is reported in *Solanaceae* with the exception of potato [31]. Indeed, the genetic structure and diversity present in *Capsicum* germplasm was only investigated using a large set of SSR markers [14, 15]. The recent whole genome sequencing of *Capsicum* [32, 33] provides a unique opportunity to estimate chromosome wide molecular diversity and precisely infer pepper population structure, enhancing the information derived from GBS data.

We used the GBS approach to identify genome-wide SNPs in a collection of 370 *Capsicum* spp. accessions, and to assess the level of genetic diversity in a subset of 222 cultivated pepper (*C. annum*) genotypes including landraces, cultivars, hybrids, breeding lines, wild and ornamental lines collected from across the world. We determined population structure and estimated genetic diversity with the long-term aim of developing a reduced subset of accessions to be exploited for future association mapping studies.

## Methods

### Plant collection

GBS was performed on a collection of *Capsicum* spp. genotypes including *C. annuum* (229), *C. frutescens* (14), *C. chinense* (59)*, C. chacoense* (13)*, C. galapagoense* (1), *C. pubescens* (12), *C. baccatum* (41), *C. praetermissum* (1). For genetic diversity and the associated population structure analysis we considered only a collection of 222 *C. annuum* accessions with different biological status: 110 landraces, 72 varieties, 15 hybrids, 13 ornamentals, 8 breeding lines and 4 wild lines (Additional file 1: Table S1). The list of non-*annuum* species we have not considered for genetic diversity analysis is reported in Additional file 1: Table S2. Accessions were sampled from 25 different countries of Europe, Asia, Africa, America and were initially retrieved from local farmers (Piemonte, Campania and Calabria regions) in Italy, associations (www.pepperfriends.com), seed companies (Nunhems, Semiorto Sementi, Esasem), research institutes (Chile Pepper Institute, Inst. Agrobiotecnology Turkey, CREA, University of Turin) and germplasm banks (Centre for Genetic Resources, CGN, Wageningen, The Netherlands, and the Leibniz-Institut für Pflanzengenetik und Kulturpflanzenforschung, IPK, Gatersleben, Germany). Subsequently, the lines were subjected to two cycles of controlled self-fertilization under glasshouse conditions at CREA-ORT. The genotypes used are characterised by a large phenotypic diversity in terms of fruit related traits (morphology, shape and colour), pungency, resistances and end-uses.

### SNP discovery by GBS

GBS involves five major steps: sample preparation, library assembly, sequencing, SNP calling and diversity analysis. Genomic DNA was extracted using the DNeasy® Plant Mini Kit (QIAGEN, Germany). DNA quality parameters as well as concentration were measured by absorbance values at 260 and 280 nm respectively, using a UV-Vis spectrophotometer (ND-1000; NanoDrop, Thermo Scientific, Wilmington, DE, USA). A trial DNA digestion was carried out using the 6-base-cutter *Hind*III. GBS was performed at the Institute of Genomic Diversity (Cornell University, Ithaca, NY, USA) as described by Elshire [17]. Genome complexity was reduced by digesting individual sample genomic DNA with *Ape*KI, a methylation sensitive restriction enzyme. The resultant fragments from all samples were directly ligated to a pair of enzyme-specific adapters, and were combined into pools. PCR amplification was carried out to generate the GBS library, which was submitted to a single Illumina HiSeq 2500 run (Illumina Inc., USA). The sequencing produced millions of reads split across multiple FASTQ files. All unique sequence tags from each sequence file were captured and then collapsed to generate a master tag file. Master tags were aligned to the reference CM334 genome available at http://peppergenome.snu.ac.kr [33] using the Burrows-Wheeler Aligner (BWA) tool (version 0.7.8-r455) with default settings. The GBS analysis pipeline implemented in TASSEL (version 3.0.166) was used to call SNPs [34]. SNP calling implemented within the TASSEL-GBS pipeline produced a raw HapMap genotypic data file. A two-step filtering procedure was used in order to filter high quality SNPs. Initial filtering was performed with settings for minimum minor allele frequency (mnMAF = 0.01), minimum taxa coverage (mnTCov = 0.1) and minimum site coverage (mnSCov = 0.8). The genotypes with a large number of missing data were filtered out based on minimum minor allele count (mnMAC = 10). SNPs that passed either the specified minimum minor allele count (mnMAC) or frequency (mnMAF), were kept for downstream analysis. Subsequently, we filtered out high quality SNP markers using TASSEL-GBS with the following parameters: minimum count 150, minimum frequency 0.01 and Maximum Frequency 1.0.

Read depth and coverage data were obtained using custom R scripts and BEDTools [35]. In order to identify the peri-centromeric regions of the 12 *Capsicum* chromosomes we used the pepper COSII genetic map [36]. For each chromosome, peri-centromeric flanking markers were selected and their position was defined from the information available at the Sol Genomics Network [37]. In Additional file 1: Table S3 the COSII markers, used to define the peri-centromeric regions, are reported. Vcf-annotate form the VCFtools (0.1.13) was used to count how many SNPs fall within coding regions. All sequences were submitted to the NCBI Short Read Archive (SRA; http://www.ncbi.nlm.nih.gov/sra/) under the accession number SRP070992.

### Genetic diversity and population structure analysis

Polymorphic Information Content (PIC), Heterozygosity (H^2^) and Gene Diversity were calculated using Power Marker software [38]. In order to investigate the population structure, assess genetic diversity and remove near-duplicates (i.e. highly similar genotypes), both parametric and non-parametric approaches were used. Population structure was determined using the parametric Bayesian model-based clustering method implemented in STRUCTURE v.2.3 (http://pritch.bsd.uchicago.edu/structure.html) [39], via the StrAuto (v0.3.1) program [40] which assigns individuals to K (i.e. the number of cluster in a sample of individuals) according to a membership coefficient (qi). For each K (from 2 to 15) ten independent runs were performed applying the admixture model (INFERALPHA = 1), with allele frequencies correlated for SNP markers (FREQSCOR = 1), 100,000 Markov Chain Monte Carlo (MCMC) repetitions, 100,000 burn-in period and RANDOMIZE = 1.

The optimal K value was determined by use of the *ad-hoc* statistic ΔK [41] estimated with the software Structure Harvester [42]. Next, all the accessions in each sub-group were subjected to a second STRUCTURE run with the parameters previously described. For each group STRUCTURE provided the average distances (expected Heterozygosity, He) between individuals in same cluster, the Fixation Index (F_ST_) as a measure of population differentiation based on molecular markers, and a membership coefficient (qi). A genotype was considered to belong to a group if its membership coefficient (qi) was ≥ 0.50 [43]. Genotypes with qi lower than 0.5 at each assigned K were considered as admixed.

Population structure was also inferred using the non parametric method implemented in the AWclust software [44–47]. The clustering procedure performs a Ward’s minimum-variance cluster analysis (R square = D^2^) based on the allele sharing distance (ASD) matrix, representing the underlying genetic distance between every pair of individuals. It calculates Gap statistic for estimating the optimal number of groups (K) based on the sample genetic relatedness [47]. LD decay was measured by plotting adjacent pair r^2^ values against the genetic distance (kb) between adjacent SNP loci, based on the coordinate system of the CM334 reference genome [33]. LD estimation was carried out by running the SNP & Variation Suite v8.4.0 (Golden Helix, Inc., Bozeman, MT, www.goldenhelix.com) [48] applying the EM method.

## Results

### SNP discovery and genetic diversity

Raw GBS data were analysed using the TASSEL-GBS pipeline to generate SNP calls [34]. Three hundred and seventy (370) samples were digested using the restriction enzyme *Ape*KI and sequenced using the Illumina HiSeq 2500 with 101 bp single-end reads. The sequencing produced a total of 867 million reads, split into four FASTQ files. All unique sequence tags from each sequence file were captured and then collapsed to generate a master tag file of a total of 7,568,894 sequences. Next, these master tags were aligned to the CM334 reference genome: 3,283,326 (43.4%) tags aligned uniquely to the reference; 743,881 (9.8%) aligned to multiple positions and 3,541,687 (46.8%) did not successfully align. Uniquely aligned tags were used for calculating the distribution of tag density at each position in the pepper genome and for SNP calling.

**Fig. 1:**
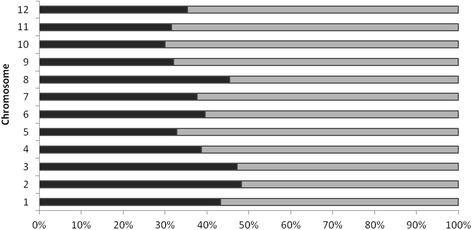
Stacked bar chart describing the distribution of master tags in gene (black) and inter-genic (grey) regions on the 12 pepper chromosomes

After mapping the master tags along the 12 *C. annuum* cv. CM334 chromosomes, the aligned sequence coordinates were extracted from the SAM/BAM files. By exploiting gene coordinates we were able to distinguish between tags overlapping genes (39%) and tags located in inter-genic regions (61%). In Fig. 1 a stacked bar chart with the percentages of gene and inter-genic tags per chromosome is reported. Notably, chromosomes 2, 3 and 8 showed a greater number of tags in gene regions than other chromosomes, while master tags preferably fall within intergenic regions in case of chromosomes 5, 9, 10 and 11. In Additional file 2: Figure S1, the distribution of tag coverage along the 12 pepper chromosomes is reported. As observed, unique tag sequences were not uniformly distributed over the 12 chromosomes, with an increase in the depth of coverage in euchromatic regions.

Tags mapped in the same physical location on the reference genome were used to identify SNPs. A total of 459,238 unfiltered SNPs were detected. Using TASSEL-GBS analysis on all samples [17, 34], and the pipeline developed at the Institute of Genomic Diversity (Cornell University), it was possible to filter out 108,591 SNPs, of which 105,187 were associated to the 222 *C. annuum* genotypes, providing an average density of one SNP every 8.7 Kb across the twelve chromosomes. Nine percent of SNPs resulted to be positioned at a distance greater than 25 Kb, while the biggest gap between SNPs was 4708 Kb on chromosome 4. In Additional file 1: Table S4 the average distance between SNPs for each chromosome is reported. A total of 35,179 SNPs (32,4%) fall within annotated exons (Table 1), affecting a total of 7477 CM334 genes. The greatest and the lowest number of SNPs within genes is on chromosomes 3 and 9, respectively. A further filtering step allowed identification of 32,950 high quality SNP markers (see Methods) which were used in genetic diversity analysis and LD estimation. Their distribution on the pepper chromosomes is reported in Additional file 2: Figure S2. SNPs were categorized according to nucleotide substitution as either transitions (C↔T or A↔G) or transvertions (A↔C, C↔G, A↔T, G↔T). As is evident from Additional file 2: Figure S3, there is a higher frequency of transitions (57.46%) than transvertions (42.54%).

**Table 1:** SNP count per chromosome

Chromosome	# SNPs	# SNPs within annotated exons	% SNPs within annotated exons	# of affected genes
1	12044	3807	31.6	816
2	11426	4466	39.1	949
3	13948	4942	35.4	1026
4	8419	2607	31.0	555
5	7886	2554	32.4	482
6	10854	3254	30.0	700
7	7754	2525	32.6	534
8	6890	2218	32.2	492
9	6428	1890	29.4	409
10	7574	2331	30.8	520
11	7058	2003	28.4	445
12	8310	2582	31.1	549
TOT	108591	35179	32.4	7477

For each chromosome it is reported the total number of polymorphisms, the number of SNPs within annotated exons and the number of affected genes

The values of Heterozygosity, PIC and the Gene Diversity index are reported in Additional file 1: Table S5. The PIC values ranges between 0.037 (chromosome 2) and 0.048 (chromosomes 9 and 10), with an average of 0.041. The mean values of the Gene Diversity index and Heterozygosity are 0.048 and 0.023, respectively. The estimate of *r* ^2^ for all pairs of linked SNP loci were used to assess the extent of LD decay. Across the genome, LD decayed rapidly (*r* ^2^ = 0.20) within 100 kb genomic regions (Additional file 2: Figure S4).

### Population structure and genetic diversity analysis

**Fig. 2:**
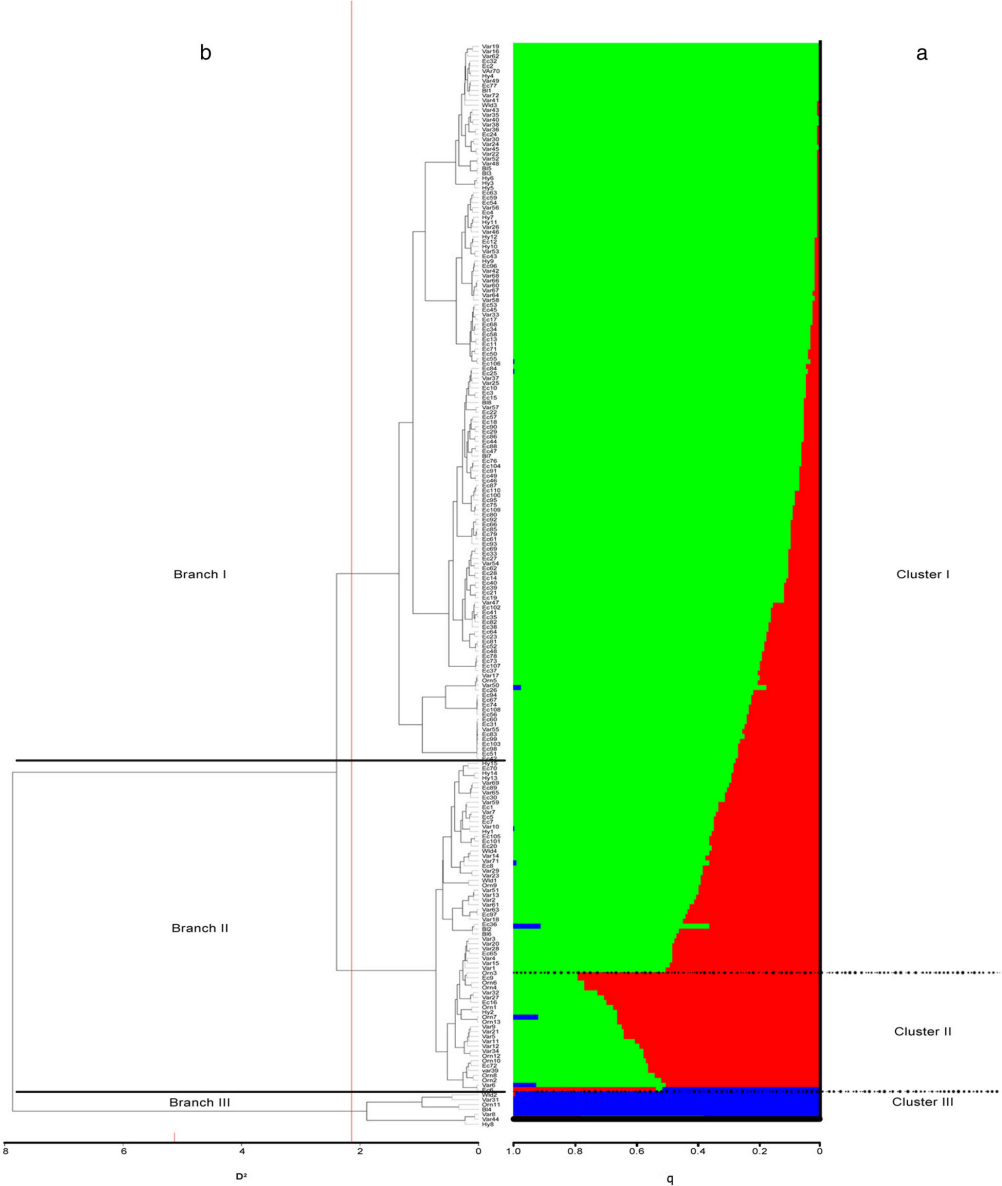
Estimate of genetic diversity in 222 *C. annuum* accessions using 32,950 SNP markers. **a** Bar-plot describing the population structure estimated by the Bayesian clustering. Each individual is represented by a thin horizontal line, which is partitioned into *K* coloured segments whose length is proportional to the estimated membership coefficient (q). The population was divided into three (K = 3) groups according to the most informative K value (see Additional file 2: Figure S5). Dashed black lines separate individuals in different clusters. **b** Dendrogram plot derived from the non-parametric hierarchical clustering. D^2^ indicates the allele sharing distance. Black continuous lines separate individuals of different sub-populations. The population was divided into three (K = 3) groups according to the most informative K value (see Additional file 2: Figure S6)

Based on 32,950 SNP loci from 222 accessions, the population structure within *C. annuum* was investigated. We ran the STRUCTURE software with *K* ranging from 2 to 15 and performed 10 independent runs for each *K.* Evanno’s test [41] was applied as a criterion to infer the most likely K value. To this end, we used the Structure Harvester software, which provided mean LnP(K) and ΔK values from K = 2 to K = 15 (Additional file 2: Figure S5). The maximum delta K was detected at K = 3 and, as a consequence, the population was divided in 3 clusters including 191, 23 and 6 accessions, respectively; 2 genotypes were classified as admixed (Fig. 2).

**Fig. 3:**
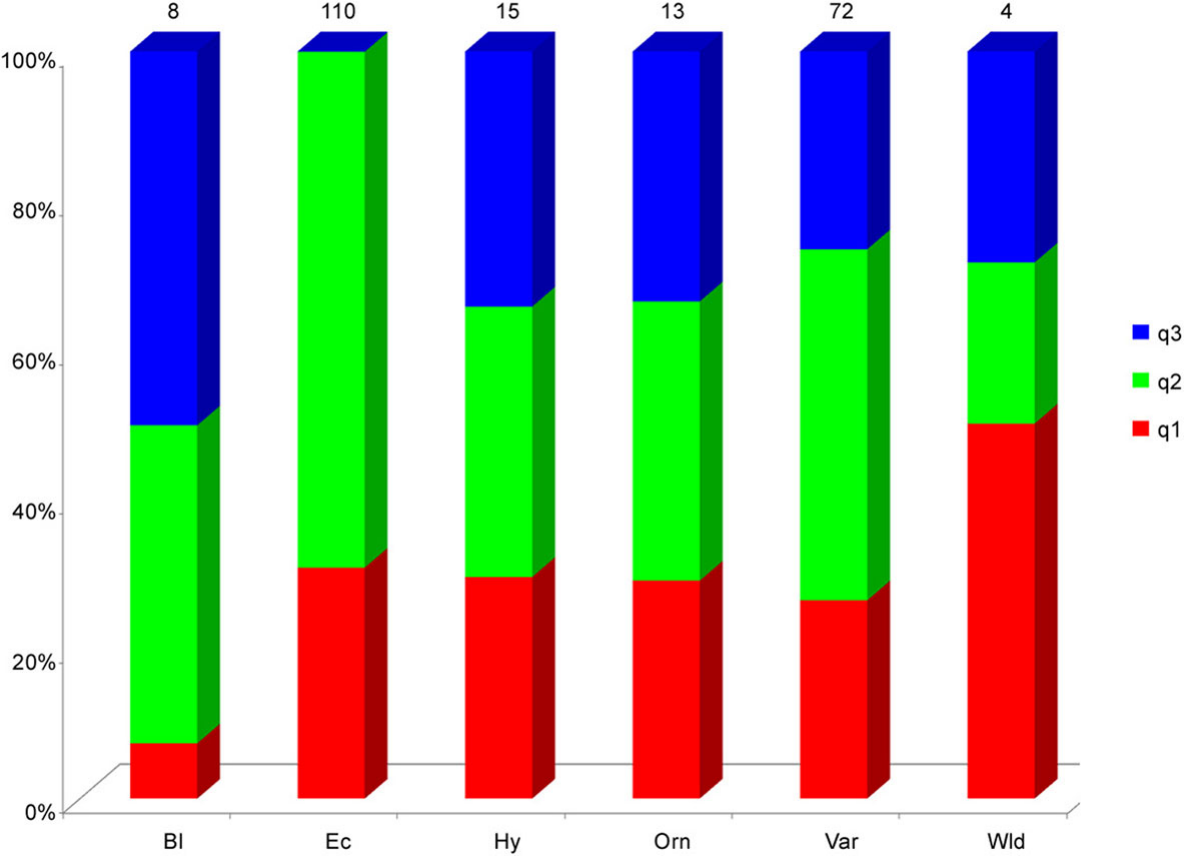
Stacked bar chart of the allele frequency (q membership coefficient) at K = 3 of groups of accessions characterised by a different biological status*.* Bl = breeding lines; Ec = ecotypes; Hy = hybrids; Orn = ornamentals; Var = varieties; Wld = wilds. The number of accessions is indicated above each bar

The majority of varieties and landraces originating from Southern and Northern Italy, Eastern Europe (mainly Hungary, Romania, Ukraine) and Turkey were assigned to cluster I (He 0.08, F_ST_ 0.85, q2 0.88). Additional genotypes from Asian countries, Mexico, and USA were included in this cluster as well. This main cluster had 86% of the genotypes, and these are characterised by a different biological status and differences in terms of fruit shape and pungency level. The accessions grouped in cluster II (He 0.16, F_ST_ 0.71, q1 0.67) included only hot peppers from various geographical areas. Finally, cluster III (He 0.48, F_ST_ 0.33, q3 0.84) grouped genotypes with different origins. Considering the average q-value at K = 3 (Fig. 3), the analysis allowed the accessions tagged as ecotypes to be distinguished from the remaining ones.

In order to validate the population structure, the AWclust hierarchical non-parametric method was also applied. The estimation of correct number of sub-populations (K) was identified using the Gap statistic, with values ranging from K = 1 to K = 15 (Additional file 2: Figure S6). The Gap statistic suggested the optimal K to be 3, corroborating the population structure obtained from the Bayesian approach. Based on this information, the dendrogram tree was cut at K = 3 to generate three major branches with 147, 68 and 7 accessions, respectively (Fig. 2). Applying this method, a better division in the geographic region of origin was observed: in particular, branch I contains Italian, Eastern European and Turkish genotypes, accessions from other world locations belong to branch II, while hot peppers with different origins are in branch III. Notably, a general overlap was observed between STRUCTURE clusters and AWClust branches: cluster I corresponding to branch I and cluster III to branch III. Conversely, branch II included a larger number of accessions compared to cluster II in STRUCTURE, merging together the whole cluster II, the admixed genotypes and 44 ones belonging to cluster I. Taking into account a minimum variance cluster <1.0 in the AWclust-derived dendrogram (Additional file 1: Table S6, Additional file 2: Figure S7), genotypes from the Balkans and Turkey tended to cluster together (A1.1.1.1a) as well as hybrids from Hungary (A1.1.1.1b). Elongated and sweet blocky types from Italy, Eastern Europe and USA (A1.1.1.2_a) clustered together, as well as sweet blocky accessions retrieved from North Italy (A1.1.1.2_b).

**Fig. 4:**
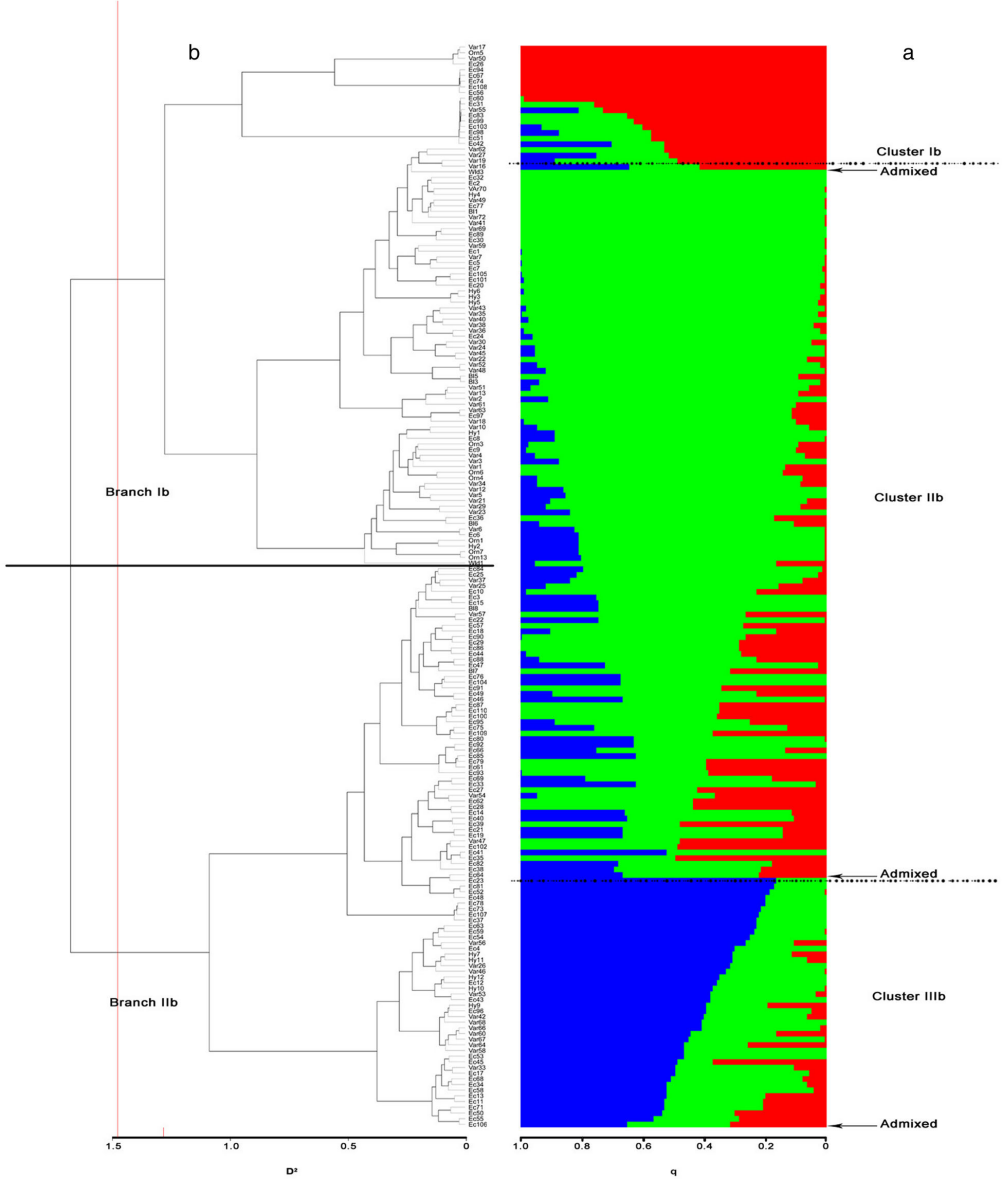
Estimate of genetic diversity in a sub-population of 191 *C. annuum* accessions using 32,950 SNP markers. **a** Bar-plot describing the population structure estimated by the Bayesian clustering. Each individual is represented by a thin horizontal line, which is partitioned into *K* coloured segments whose length is proportional to the estimated membership coefficient (q). The population was divided into three (K = 3) groups according to the most informative K value (see Additional file 2: Figure S8). Dashed black lines separate individuals in different clusters. **b** Dendrogram plot derived from the non-parametric clustering. D^2^ indicates the allele sharing distance. The population was divided into two (K = 2) groups according to the most informative K value (see Additional file 2: Figure S9). Black continuous lines separate individuals of different sub-populations

**Fig. 5:**
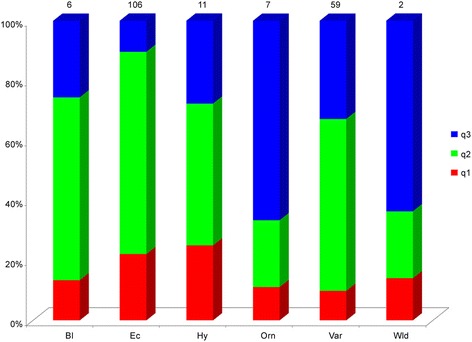
Stacked bar chart of the allele frequency (q membership coefficient) at K = 3 of groups of accessions characterised by a different biological status*.* Bl = breeding lines; Ec = ecotypes; Hy = hybrids; Orn = ornamental; Var = varieties; Wld = wilds. The number of accessions is indicated above each bar

A main cluster of chili peppers from the Calabria Region in Southern Italy comprised conical and cherry types (A1.1.2.1.1_a - A1.1.2.1.1_b). A further large group included sweet accessions from different regions of Southern Italy (A1.1.2.1.2) while a few other spicy genotypes were in group A.1.1.2.2. A mixture of conical Italian ecotypes were in subgroup A1.2 (A1.2.1_a, A1.2.1_b, A1.2.2), two cherry-like genotypes were identified in subgroup A1.2.1_b. In group A2 were all chili peppers with different origins and most of the ornamental accessions included in the collection. No blocky or elongated types were within this cluster. A large group included Mexican, Italian, Spanish (A2.1.1.1_a, A2.1.1.1_b), while the other accessions clustered together in several small subgroups. An additional heterogeneous group (A2.2.1) included several conical genotypes from 13 countries. Considering the large number of genotypes belonging to cluster I we hypothesized a large variability within this sub-population. For this reason, a second round of STRUCTURE and AWclust was performed considering only the genotypes belonging to cluster I. As described above, the maximum delta K was again inferred at K = 3 (Additional file 2: Figure S8) and the population was accordingly divided in three sub-populations (Fig. 4), comprising 20, 122 and 35 accessions, respectively; the remaining 14 genotypes were classified as admixed. Conical hot peppers mainly from Italy were grouped in the cluster Ib (He 0.12, F_ST_ 0.48, q1 0.80) this cluster also includes three genotypes from Spain, Brazil and Turkey. Cluster IIb (He 0.05, F_ST_ 0.59, q2 0.80) comprises sweet blocky types mainly from Italy, Turkey and Hungary as well as hot pepper ecotypes from Southern Italy. Cluster IIIb (He 0.06, F_ST_ 0.027, q3 0.67) includes varieties and ornamentals from Eastern Europe, Asia, and America. Based on the average q-value, ornamental and wild accessions showed similar allele frequencies, while the remaining genotypes present a clearly distinguishable genetic structure (Fig. 5).

AWclust-based hierarchical clustering defined two main sub-population according to the Gap statistic with values ranging from K = 1 to K = 15 (Additional file 2: Figure S9). The first branch (Ib) includes most of the Italian accessions and few genotypes of the Mediterranean area for a total of 115 individuals. All blocky and roundish fruits, including accessions from the US (Yolo Wonder, Yolo Y, California Wonder, Chocolate Beauty), clustered within this group. The second branch (IIb) includes 62 conical chili pepper genotypes from different countries.

By comparing STRUCTURE clusters versus AWclust branches it was possible to observe that all the accessions in the cluster Ib and IIIb were part of the branch Ib and IIb, respectively. The accessions belonging to the cluster IIb and the admixed were distributed in both branches.

Finally, we selected a reduced number of accessions from the original collection that represent most of the genetic variation with minimum redundancy. To this end, in case of accessions belonging to cluster I we used the ASD matrix and fixed the R squared (D^2^) value equal to 0.08 to consider one accession a good surrogate of each other. In this way we reduced the dataset from 191 to 117 accessions. The latter must be added to accessions in cluster II (23), III (6) and in the admixed group (2), all characterised by a wide genetic variability. In conclusion, the reduced set is composed of 148 accessions, representing, with minimum repetitiveness, the genetic diversity of the *Capsicum annuum* species in this collection.

## Discussion

### Genotyping by sequencing

Genotyping by sequencing is a high-throughput and low-cost technology used in several crop species to facilitate the identification and selection of target plants to be used in breeding programs [18]. Here we present an assessment of the genetic diversity in a collection of *C. annuum* including mostly Italian genotypes by using the GBS approach. As far as we know, this is the first report combining genome-wide genetic marker discovery and genotyping using next-generation sequencing revealing the genetic diversity and population structure in pepper.

GBS was performed on a large collection including 370 accessions of *Capsicum* spp. The *Ape*KI enzyme was used to reduce genome complexity and a high number of master tags was produced, of which only 43.4% aligned with the CM334 reference genome [33]. A possible reason that the majority of master tags did not successfully align to the reference genome was the very stringent parameters used by the Burrows-Wheeler Aligner (BWA) tool, in order to minimize multiple alignments. Indeed, it is very likely that most of the reads did not exceed the edit-distance threshold value because master tags can include sequencing errors or nucleotide polymorphisms. Indeed, a third of the accessions in the *Capsicum* collection are not *C. annuum* therefore different from reference genome; as a consequence nucleotide diversity among pepper species is expected. Finally, a further explanation is that some of the reads could derive from DNA segments not represented on the reference genome or belonging to cytoplasmic organelles. Lowering the alignment threshold would allow nucleotide polymorphism to be more tolerated but, by contrast, this procedure would increase the number of false positive due to incorrect alignments. Based on the compositional properties of the reference genome (characterised by a great accumulation of repetitive sequences, accumulated primarily in heterochromatic regions) [33, 49], we selected the restriction enzyme *Ape*KI because it is partially sensitive to methylation and cuts retrotransposons rarely. As a consequence, *Ape*KI digestion preferentially generates fragments from “low-copy” genomic regions [17, 50]. As shown in the Additional file 2: Figure S1 read depth varies considerably between heterochromatic and euchromatic regions in each chromosome. Read distribution is not uniform and the depth of coverage was larger in euchromatic regions. This is in accordance with the properties of the restriction enzyme *Ape*KI. Using the genotype CM334 as reference, SNP calling generated 32,950 high quality SNPs associated to 222 *C. annuum* genotypes. The identification of a relatively high frequency of SNPs showing transition substitutions (57%) over transvertions is in agreement with previous genome-wide SNP discovery studies in crops [51, 52]. This phenomenon known as ‘transition bias’ was previously reported in rice [53] and maize [53, 54], and is attributed to a higher frequency of transitional mutations over transvertions because of conformational advantage in case of mis-pairing, and better tolerance of transitions during natural selection, because transitions are more likely to conserve protein structure than transvertions [55].

Based on SNP markers generated by GBS, the level of heterozygosity in the population under investigation was very low and comparable to previous studies in *C. annuum* based on SSR markers [15, 56]. This low value is expected and it can be ascribed to the highly inbreeding nature of both domesticated and wild *C. annuum* accessions.

PIC values and Gene Diversity index we calculated are low in comparison to the values derived from studies using SSR markers. These discrepancies can be explained considering the nature of the different types of markers; SSRs are multi-allelic and more polymorphic than SNP markers which are bi-allelic.

### Genetic diversity and geographic distribution

The analysed germplasm represents a mixture of genotypes including landraces, cultivars, hybrids, breeding lines, ornamentals and wild lines from 25 different regions. Of 222 accessions, 98% were the cultivated species (*C. annuum*), while the remaining 2% comprised wild types (*C. annuum* var *glabriusculum*). We determined the population structure using two approaches. Based on Bayesian model-based clustering and Hierarchical clustering analysis, it was possible to subdivide the collection into 3 major clusters according to the maximum delta K. These analyses provided a biological interpretation of the sub-population structure; in fact, observing the subgroups within the obtained clusters (Additional file 2: Figure S7; Additional file 1: Table S6), it was possible to distinguish the accessions considering both geographical origin and fruit characteristics. This observation agrees with the report of Nicolai [56], where a combination of local selections and area confinement influenced the diversification. Also, different routes of trade may have influenced the distribution of pepper genetic material, particularly for landraces where it is hypothesized that trade routes by sea were preferred [57]. This could explain, for instance, why Northern and Southern Italian ecotypes were quite distinguishable, while accessions retrieved from countries bordering the Black sea (Turkey, Ukraine and Hungary) as well as those from Spain and Western Africa were closely related. Clustering based on GBS data allowed accessions to be clearly separated based on fruit-related features: conical types clustered close to each other, and the elongated and blocky types tend to form separate groups. This could explain the similarity of the common blocky American types, such Yolo Wonder and Yolo Y, to Italian sweet blocky peppers. By contrast, round types and cherries were grouped more according to their geographical origin than on the basis of fruit shape, indeed, they cluster with conical genotypes. Observing accession distribution across clusters, it was possible to consider pungency as an additional parameter influencing the diversification. Sweet and spicy genotypes are in different clusters. In some cases, accessions clustered separately even though from the same geographical region (i.e. groups A1.1.2.1.2 and A1.1.2.2). In other cases, sweet genotypes clustered with chili pepper based on geographical origin (i.e. group A1.1.2.1.1_a). This trend was particularly observed in landraces. In any case, in the present study, the grouping of the accessions is mediated by fruit shape, considering, as example, that all blocky types are sweets. The groups A2 and B contained only spicy genotypes (except Ec72 and Var39); the accessions included in these groups were much more spicy than those within group A1 (Tripodi et al., unpublished data). The collection analysed included four accessions of *C. annuum* var. *glabriusculum.* This species, commonly named “chiltepin” or “pequin”, is characterised by small fruits (about 0.5 cm diameter) and bushy plants, and it is considered as the wild parent of cultivated *C. annuum* [58]. These four accessions did not cluster, being distributed in all the identified clusters. This finding is in agreement with previous reports [56] where the distribution of this species in several clusters is highlighted. Probably, the geographical distribution of chiltepin accessions and their large within accessions genetic variability [59], could have affected the distribution of these wild genotypes among the clusters identified in the present study. The Bayesian analysis has shown that the population is structured in a few main groups, even considering higher K level (data not shown). Similar studies highlighted the low number of groups within *C. annuum*: as an example, 935 *C. annuum* genotypes were subdivided in 3 clusters [56], while maximum ΔK value of 2 was observed in genetic diversity analysis involving *annuum* and *non-annuum* germplasm accessions [15, 60].

The subsequent division of cluster I allowed a deep investigation of the genetic structure of the sub-population consisting of 191 accessions. The three different clusters (Additional file 1: Table S7) identified by the Bayesian approach reflect the distribution described above, in particular chili pepper ecotypes from the Calabria Region (q2) were quite distinguishable from other chilli genotypes from other regions, highlighting the effect of local selection. In addition, the Mediterranean accessions were quite distinct from those from other locations. The Hierarchical method allowed identification of two main branches separating most of the Italian accessions from those of other origins. In both analyses, ornamentals and most varieties from Asia made a clearly distinct group. Among the accessions of different biological status, the ecotypes showed distinct allele frequencies in both the two rounds of clustering (Figs. 3 and 5). The particular allele frequency values within this group of genotypes could be due to farmers’ selection practices and/or may be ascribed to specific genotype x environment interactions.

A rapid decay of LD was observed in the 222 accessions. A previous study on a collection of 96 *Capsicum* individuals genotyped by 176 SSRs reports a mean LD of 32.17 Mb [61]. Several factors are thought to influence LD in plants, such as genetic drift, mating system, high levels of selfing and the history of selection [62]. In well-studied crops, coalescent simulations report a LD decay in outcrossing species of 500 bp while for highly selfing species the LD may extend to 10 kb [62]. Maize and *Arabidopsis* have a 250 fold difference in LD decay, several kilobases in the former and within hundreds of kilobases in the latter [62]. Moreover, in maize LD decay is within 1 kb in landraces; it is extends to 2 kb in diverse inbred lines and goes up to 100 kb in commercial elite inbred lines [63]. In a study of four loci in barley, the LD extends up to hundreds of kb in elite lines while decays to less than 1 kb for the same region in wild lines [63]. The majority of the accessions analysed in the present study are ecotypes and this could be reflected in the estimation of the LD decay [64, 65]. The effect of LD decay in the pepper collection under investigation and its implication in marker-trait associations, will be refined in further GWAS studies.

A core set of 148 accessions of *C. annuum* was built with the objective to optimize the contribution of the *C. annuum* clusters and maximize the genetic diversity. This reduced collection represents the genetic diversity of the larger collection we analysed, maintaining the different geographical origin and fruit-related features. In pepper, a small number of core collections are already developed and published based on phenotypic traits and cluster analysis [56, 66]. We have established a collection of cultivated pepper, using GBS data, confirming that genotyping by sequencing can be used to accurately estimate diversity in more diverse sets of germplasm [67].

## Conclusion

Genotyping by sequencing data has proved useful and reliable for the identification of high quality SNPs we exploited for investigating genetic diversity and defining the population structure of a *Capsicum annuum* collection. The combination of Bayesian and Hierarchical clustering tools proved to be effective in elucidating population genetic structure of pepper genotypes since the two methods corroborate each other very well. It is clear that the distribution of the genotypes within clusters reflects both geographical origin and fruit-related features, that we believe to be the main parameters influencing the observed diversification. Finally, this analysis allowed removal of near duplicates from the collection obtaining a subset representing the majority of *C. annuum* genetic variation with minimum redundancy. This work is a first step towards future genome-wide association mapping studies and the identification of SNP markers able to enhance the precision breeding for horticultural traits in cultivated pepper.

## Additional files

Additional file 1: Table S1. List of the *Capsicum* accessions used for SNP discovery and population structure. **Table S2.** List of additional non-annuum accessions genotyped through GBS. **Table S3.** COSII markers used for the identification of the peri-centromeric regions for each of the 12 pepper chromosomes. **Table S4.** Average SNP density on each chromosome, considering 105,187 SNPs detected for *C. annuum*. **Table S5.** Gene diversity, Heterozygosity and PIC evaluated across the 12 pepper chromosomes. **Table S6.** Distribution of the 222 accessions according to Hierarchical cluster analysis and Bayesian model-based clustering (K=3). **Table S7.** Clustering of 191 genotypes selected from accessions grouped in cluster I (Fig. 2), based on Hierarchical cluster analysis (K=2) and Bayesian model-based clustering (K=3). (XLS 2.14 mb)
Additional file 2: Figure S1. Bar charts illustrating the distribution of master tag coverage at each position, along the 12 pepper chromosomes. The peri-centromeric region for each chromosome is indicated in grey and has been identified using the markers listed in the Additional file 1: Table S3. **Figure S2.** Bar chart describing the distribution of the 32,950 SNPs on the 12 pepper chromosomes. **Figure S3.** Bar chart describing the distribution of SNP types divided according to nucleotide substitution as transitions (black) and transvertions (grey). **Figure S4.** Scatter plot of linkage disequilibrium decay (r2) against the genetic distance for linked SNP throughout the CM334 pepper reference genome. **Figure S5.** Evaluation of the best grouping number (*K*) of the Bayesian clustering analysis using the Evanno’s method. a) Plot of mean likelihood L(K) and variance for 10 independent runs for each value of K for K = 2–15. b) Evanno’s plot generated by STRUCTURE HARVESTER for the detection of the true number of clusters (the most likely value of K). The highest value was at K = 3, indicating that the 222 accessions likely form 3 sub-populations. **Figure S6.** Gap statistic plots for the dataset including 222 pepper accessions. The number of inferred Ks ranging from 1 to 15 are shown in the graph. a) The blue and red curves are the estimated expectation of log (Wk) and the observed log (Wk), respectively. b) The x-axis represents different possible Ks (K3 is the best value) and y-axis represent the gap value. **Figure S7.** Hierarchical clustering (K = 3) of 222 *C. annuum* accessions and derived subgroups obtained at minimum variance cluster <0.1, using the AWclust software. **Figure S8.** Evaluation of the best grouping number (*K*) of the Bayesian clustering analysis performed on 191 *C. annuum* accessions using the Evanno’s method. a) Plot of mean likelihood L(K) and variance for 10 independent runs for each value of K for K = 1–15. b) Evanno’s plot generated by STRUCTURE HARVESTER for the detection of the true number of clusters (the most likely value of K). The highest value was at K = 3, indicating that the 191 accessions included in cluster I (Fig. 2) likely form 3 sub-populations. **Figure S9.** Gap statistic plots for the dataset including the 191 pepper accessions belonging to cluster I (Fig. 2). The number of inferred Ks ranging from 1 to 15 are shown in the graph. a) The blue and red curves are the estimated expectation of log (Wk) and the observed log (Wk), respectively. b) The x-axis represents different possible Ks (K2 is the best value) and y-axis represent the gap value. (PDF 3470 kb)

## Abbreviations

AFLP: Amplified Fragment Length Polymorphism
ASD: Allele sharing distance matrix
BC: Backcross populations
BWA: Burrows-Wheeler Aligner
F_ST_: Fixation Index
GBS: Genotyping by sequencing
LD: Linkage disequilibrium
MCMC: Markov Chain Monte Carlo
mnMAC: Minimum minor allele count
mnMAF: Minimum minor allele frequency
mnSCov: Minimum site coverage
mnTCov: Minimum taxa coverage
NGS: Next Generation Sequencing
PIC: Polymorphic Information Content
RILs: Recombinant inbred lines
SNP: Single nucleotide polymorphism
SSR: Simple Sequence Repeat
