# Supplementary material for: Genome-wide SNP discovery and population structure analysis in pepper (Capsicum annuum) using genotyping by sequencing
Source: BMC Genomics. 2016 Nov 21;17:943. doi: 10.1186/s12864-016-3297-7 (PMC5117568; doi:10.1186/s12864-016-3297-7)
Supplement: Additional file 2: Figure S1. — Bar charts illustrating the distribution of master tag coverage at each position, along the 12 pepper chromosomes. The peri-centromeric region for each chromosome is indicated in grey and has been identified using the markers listed in the Additional file 1: Table S3. Figure S2. Bar chart describing the distribution of the 32,950 SNPs on the 12 pepper chromosomes. Figure S3. Bar chart describing the distribution of SNP types divided according to nucleotide substitution as transitions (black) and transvertions (grey). Figure S4. Scatter plot of linkage disequilibrium decay (r2) against the genetic distance for linked SNP throughout the CM334 pepper reference genome. Figure S5. Evaluation of the best grouping number (K) of the Bayesian clustering analysis using the Evanno’s method. a) Plot of mean likelihood L(K) and variance for 10 independent runs for each value of K for K = 2–15. b) Evanno’s plot generated by STRUCTURE HARVESTER for the detection of the true number of clusters (the most likely value of K). The highest value was at K = 3, indicating that the 222 accessions likely form 3 sub-populations. Figure S6. Gap statistic plots for the dataset including 222 pepper accessions. The number of inferred Ks ranging from 1 to 15 are shown in the graph. a) The blue and red curves are the estimated expectation of log (Wk) and the observed log (Wk), respectively. b) The x-axis represents different possible Ks (K3 is the best value) and y-axis represent the gap value. Figure S7. Hierarchical clustering (K = 3) of 222 C. annuum accessions and derived subgroups obtained at minimum variance cluster <0.1, using the AWclust software. Figure S8. Evaluation of the best grouping number (K) of the Bayesian clustering analysis performed on 191 C. annuum accessions using the Evanno’s method. a) Plot of mean likelihood L(K) and variance for 10 independent runs for each value of K for K = 1–15. b) Evanno’s plot generated by STRUCTURE HARVESTER for the detection of the true number of [file 12864_2016_3297_MOESM2_ESM.pdf]

Additional file 2

GENOME-WIDE SNP DISCOVERY AND POPULATION STRUCTURE ANALYSIS IN PEPPER (*CAPSICUM ANNUUM*) USING GENOTYPING BY SEQUENCING

Authors:
Francesca Taranto, Nunzio D'Agostino, Barbara Greco, Teodoro Cardi, Pasquale Tripodi

Corresponding author. Pasquale Tripodi, pasquale.tripodi@crea.gov.it

This PDF file includes 9 supplementary Figures:

Figure S1. Bar charts illustrating the distribution of master tag coverage at each position, along the 12 pepper chromosomes. The peri-centromeric region for each chromosome is indicated in grey and has been identified using the markers listed in the Additional file 1: Table S3.

Figure S2: Bar chart describing the distribution of the 32,950 SNPs on the twelve pepper chromosomes.

Figure S3: Bar chart describing the distribution of SNP types divided according to nucleotide substitution as transitions (black) and transvertions (grey).

Figure S4: Scatter plot of linkage disequilibrium decay (r2) against the genetic distance for linked SNP throughout the CM334 pepper reference genome.

Figure S5: Evaluation of the best grouping number (*K*) of the Bayesian clustering analysis using the Evanno's method. a) Plot of mean likelihood L(K) and variance for 10 independent runs for each value of K for K=2-15. b) Evanno's plot generated by STRUCTURE HARVESTER for the detection of the true number of clusters (the most likely value of K).The highest value was at K=3, indicating that the 222 accessions likely form 3 sub-populations.

Figure S6 Gap statistic plots for the dataset including 222 pepper accessions. The number of inferred Ks ranging from 1 to 15 are shown in the graph. a) The blue and red curves are the estimated expectation of log (Wk) and the observed log (Wk), respectively. b) The x-axis represents different possible Ks (K3 is the best value) and y-axis represent the gap value.

Figure S7: Hierarchical clustering (K=3) of 222 *C. annuum* accessions and derived subgroups obtained at minimum variance cluster <0.1, using the AWclust software.

Figure S8: Evaluation of the best grouping number (*K*) of the Bayesian clustering analysis performed on 191 *C. annuum* accessions using the Evanno's method. a) Plot of mean likelihood L(K) and variance for 10 independent runs for each value of K for K=1–15. b) Evanno's plot generated by STRUCTURE HARVESTER for the detection of the true number of clusters (the most likely value of K). The highest value was at K=3, indicating that the 191 accessions included in cluster I (Figure 2) likely form 3 sub-populations.

Figure S9: Gap statistic plots for the dataset including the 191 pepper accessions belonging to cluster I (Figure 2). The number of inferred Ks ranging from 1 to 15 are shown in the graph. a) The blue and red curves are the estimated expectation of log (Wk) and the observed log (Wk), respectively. b) The x-axis represents different possible Ks (K2 is the best value) and y-axis represent the gap value.

Figure S1

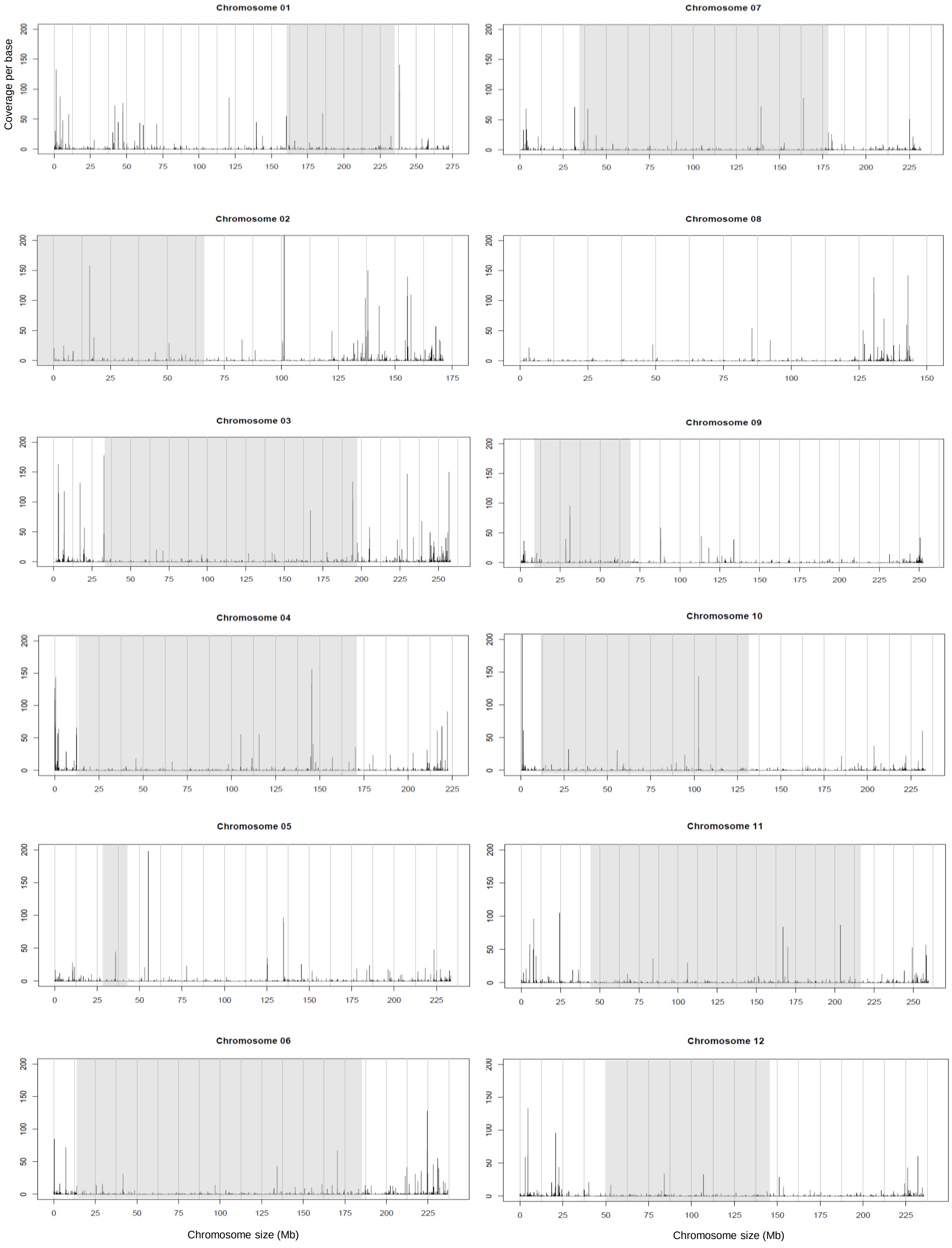
Chromosome 01
Chromosome 07
Chromosome 02
Chromosome 08
Chromosome 03
Chromosome 09
Chromosome 04
Chromosome 10
Chromosome 05
Chromosome 11
Chromosome 06
Chromosome 12
Coverage per base
Chromosome size (Mb)
Chromosome size (Mb)

Figure S2

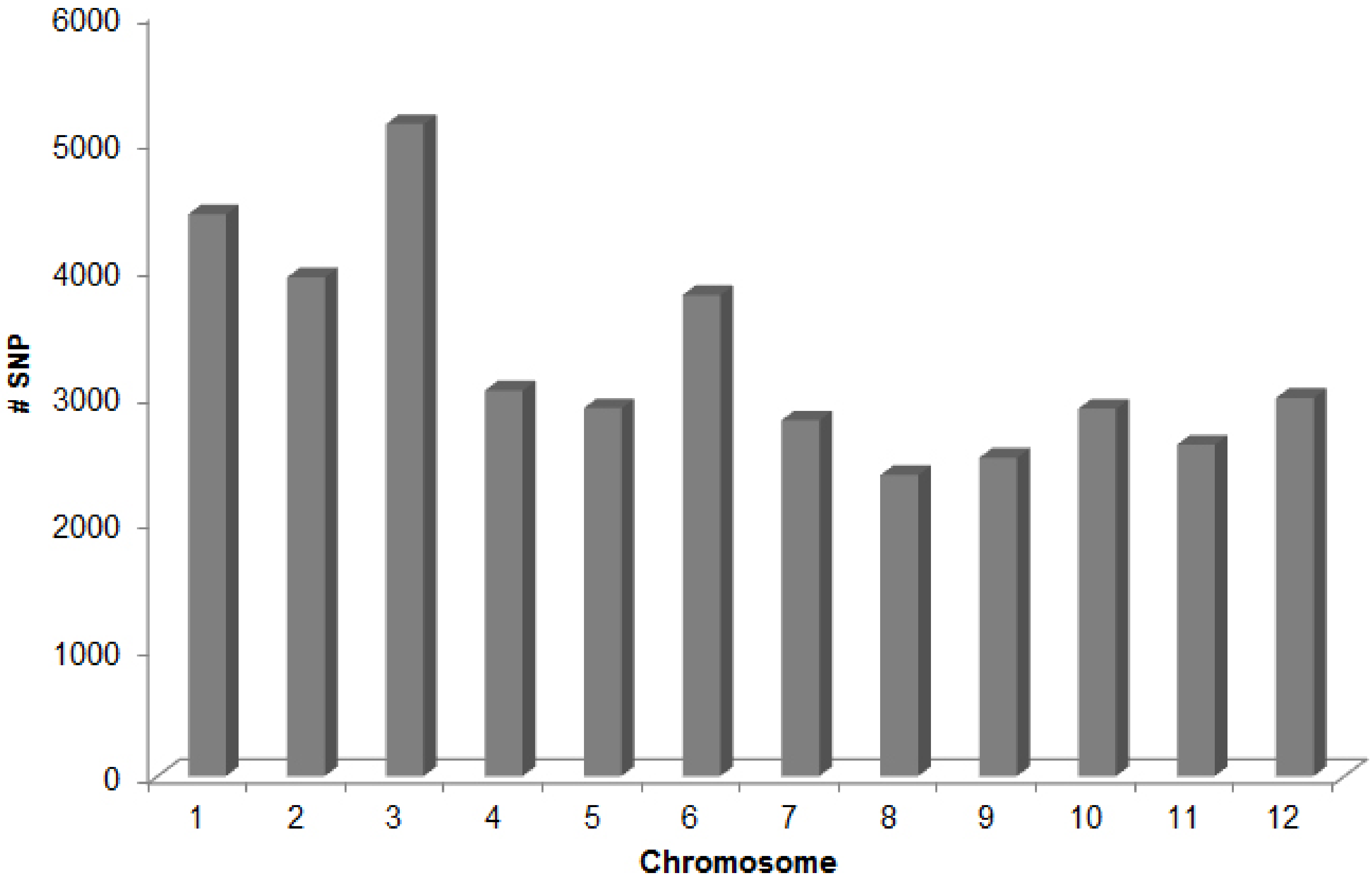

6000
5000
4000
3000
2000
1000
0
SNP
1
2
3
4
5
6
7
8
9
10
11
12
Chromosome

Figure S3

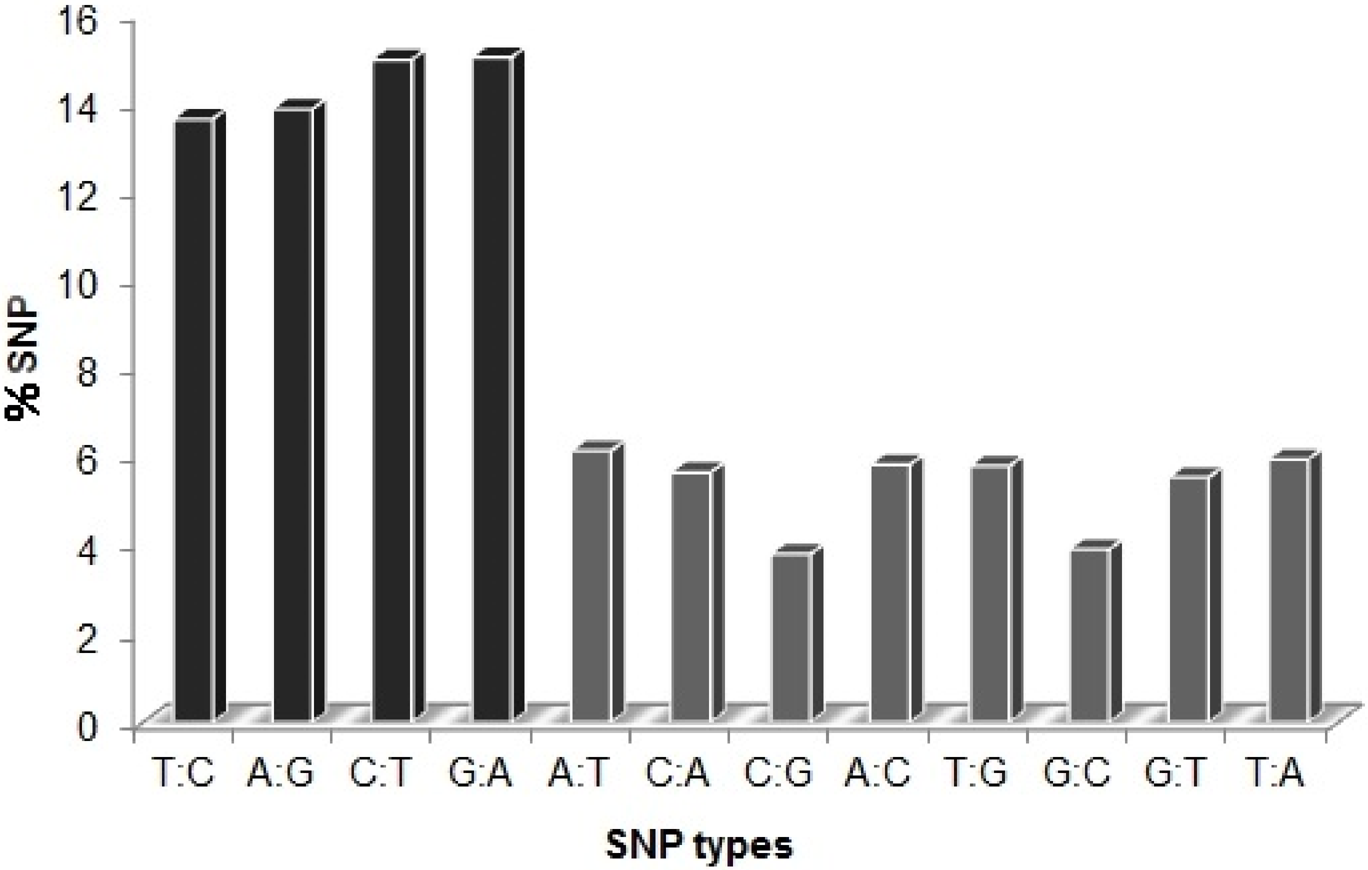

Figure S4

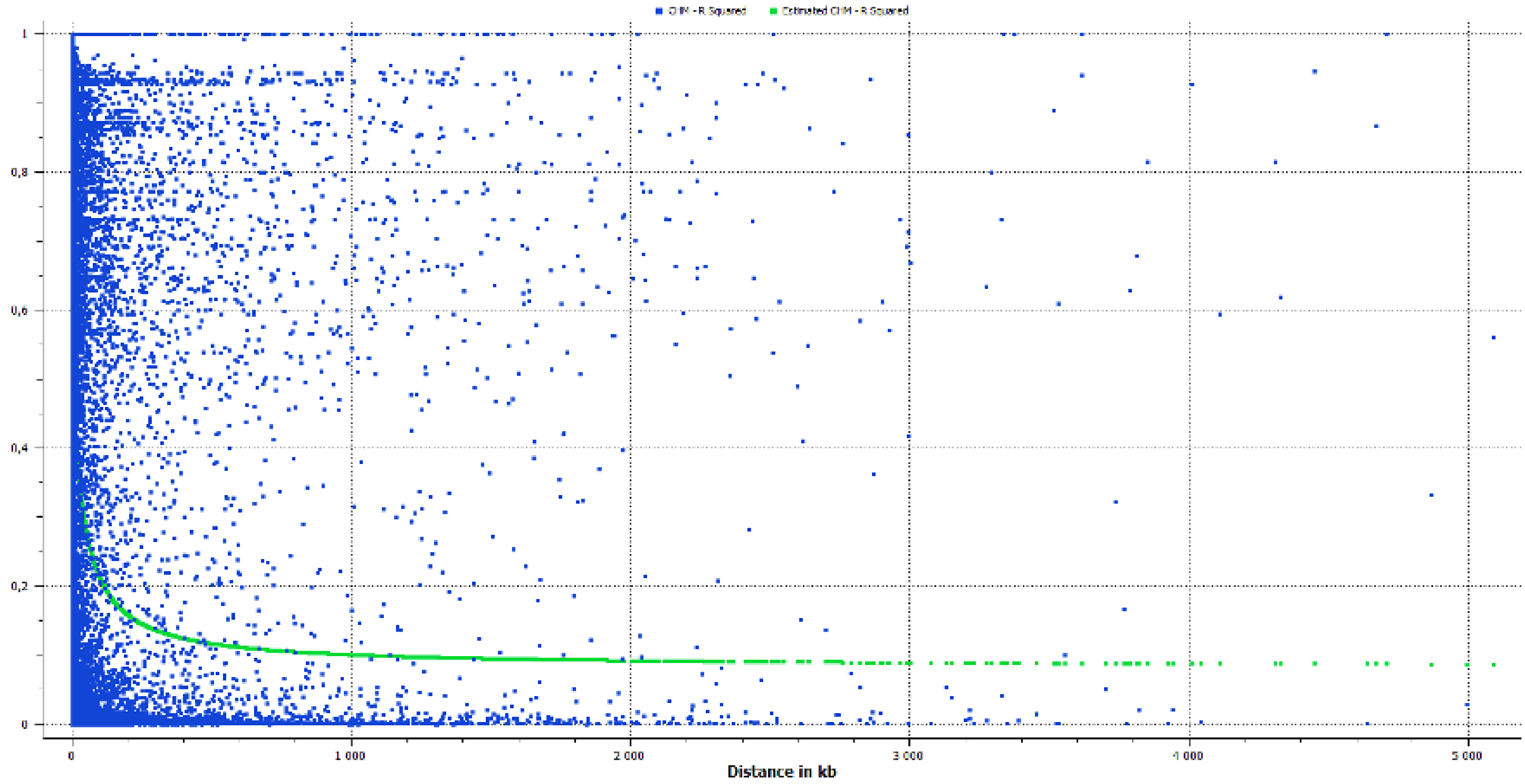

Figure S5

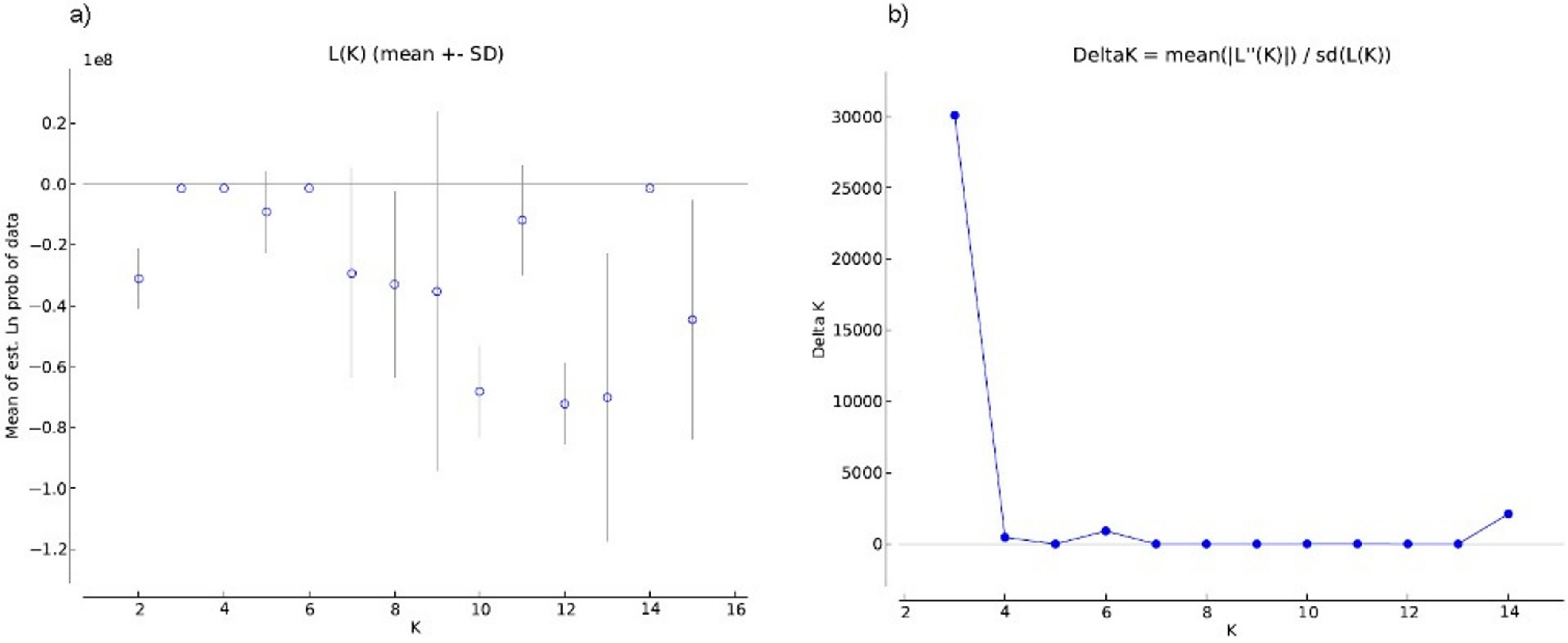
a)
L(K) (mean +- SD)
1e8
0.2
0.0
−0.2
−0.4
−0.6
−0.8
−1.0
−1.2
Mean of est. Ln prob of data
2
4
6
8
10
12
14
16
K
b)
DeltaK = mean(|L''(K)|) / sd(L(K))
30000
25000
20000
15000
10000
5000
0
Delta K
2
4
6
8
10
12
14
K

Figure S6

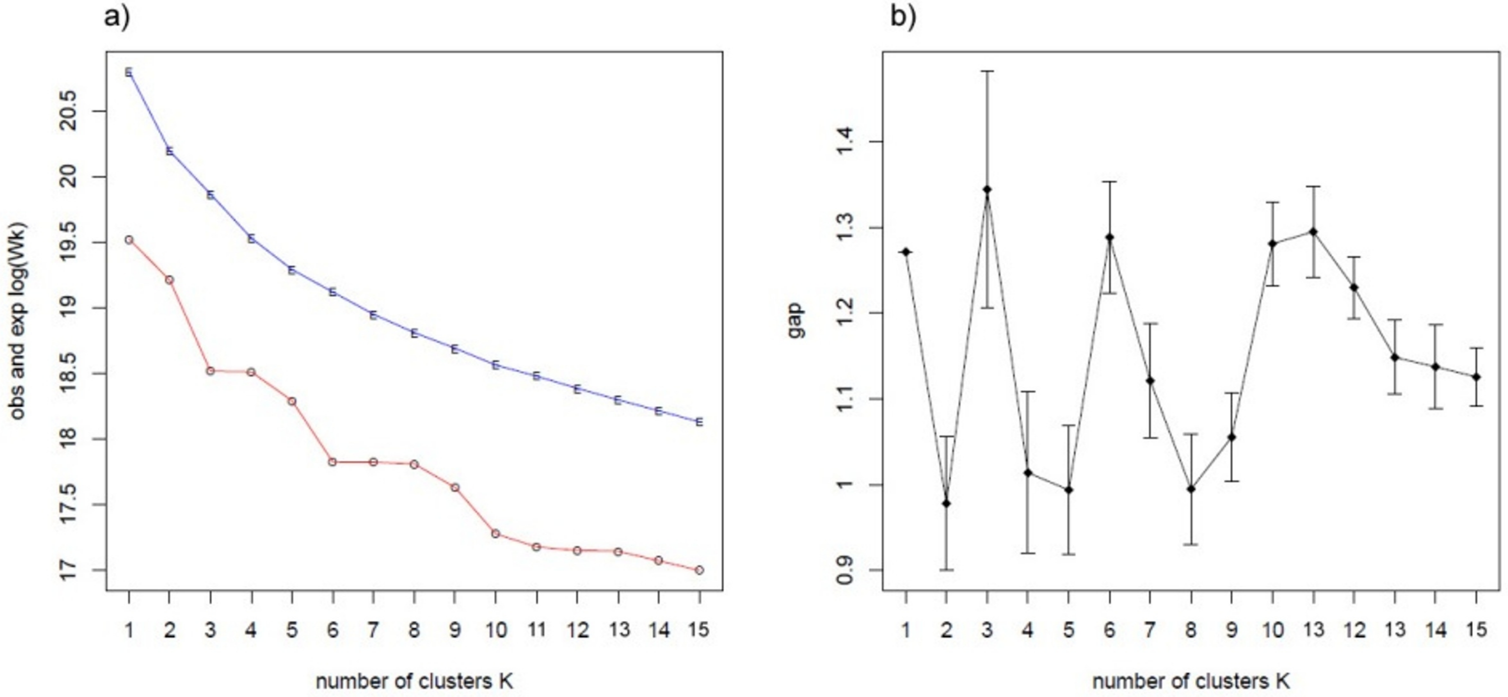
a)
obs and exp log(Wk)
17
17.5
18
18.5
19
19.5
20
20.5
1 2 3 4 5 6 7 8 9 10 11 12 13 14 15
number of clusters K
b)
gap
0.9
1
1.1
1.2
1.3
1.4
1 2 3 4 5 6 7 8 9 10 13 12 13 14 15
number of clusters K

Figure S7

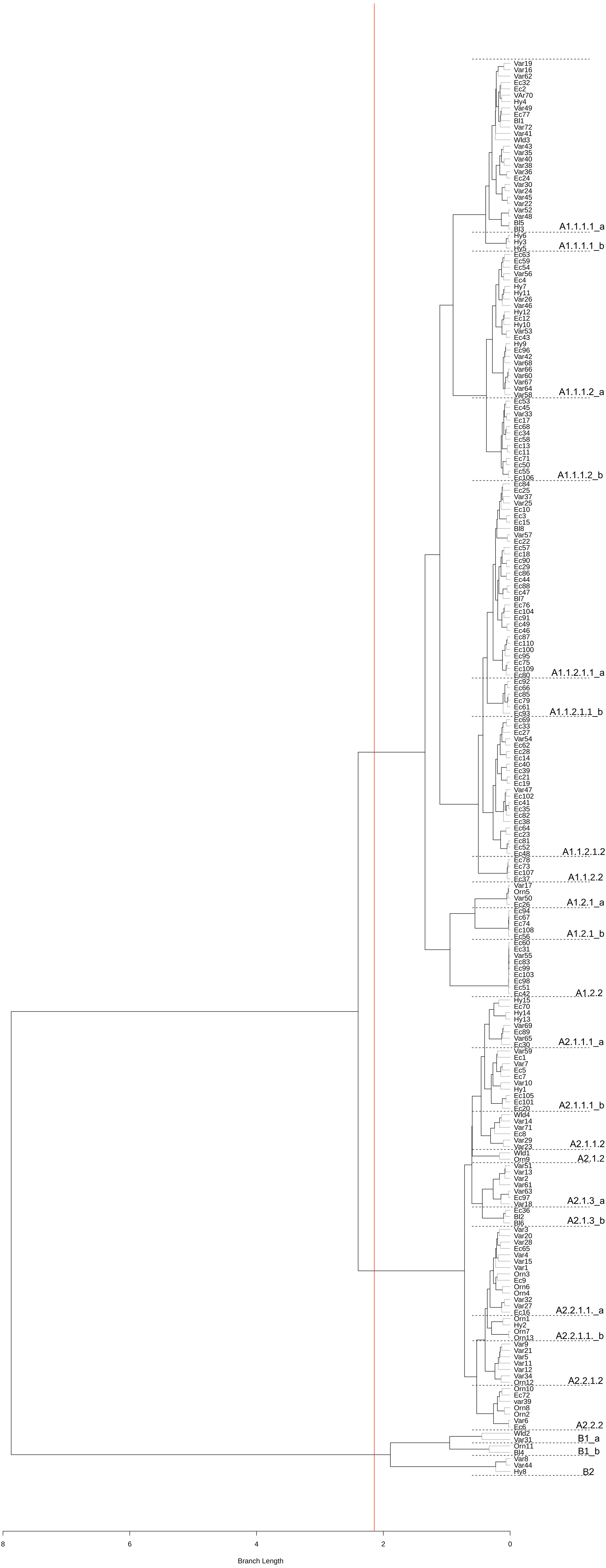

Var19
Var16
Var62
Ec32
Ec2
VAr70
Hy4
Var49
Ec77
Bl1
Var72
Var41
Wld3
Var43
Var35
Var40
Var38
Var36
Ec24
Var30
Var24
Var45
Var22
Var52
Var48
Bl5
Bl3
A1.1.1.1_a
Hy6
Hy3
Hy5
A1.1.1.1_b
Ec63
Ec59
Ec54
Var56
Ec4
Hy7
Hy11
Var26
Var46
Hy12
Ec12
Hy10
Var53
Ec43
Hy9
Ec96
Var42
Var68
Var66
Var60
Var67
Var64
Var58
A1.1.1.2_a
Ec53
Ec45
Var33
Ec17
Ec68
Ec34
Ec58
Ec13
Ec11
Ec71
Ec50
Ec55
Ec106
A1.1.1.2_b
Ec84
Ec25
Var37
Var25
Ec10
Ec3
Ec15
Bl8
Var57
Ec22
Ec57
Ec18
Ec90
Ec29
Ec86
Ec44
Ec88
Ec47
Bl7
Ec76
Ec104
Ec91
Ec49
Ec46
Ec87
Ec110
Ec100
Ec95
Ec75
Ec109
Ec80
A1.1.2.1.1_a
Ec92
Ec66
Ec85
Ec79
Ec61
Ec93
A1.1.2.1.1_b
Ec69
Ec33
Ec27
Var54
Ec62
Ec28
Ec14
Ec40
Ec39
Ec21
Ec19
Var47
Ec102
Ec41
Ec35
Ec82
Ec38
Ec64
Ec23
Ec81
Ec52
Ec48
A1.1.2.1.2
Ec78
Ec73
Ec107
Ec37
A1.1.2.2
Var17
Orn5
Var50
Ec26
A1.2.1_a
Ec94
Ec67
Ec74
Ec108
Ec56
A1.2.1_b
Ec60
Ec31
Var55
Ec83
Ec99
Ec103
Ec98
Ec51
Ec42
A1,2.2
Hy15
Ec70
Hy14
Hy13
Var69
Ec89
Var65
Ec30
A2.1.1.1_a
Var59
Ec1
Var7
Ec5
Ec7
Var10
Hy1
Ec105
Ec101
Ec20
A2.1.1.1_b
Wld4
Var14
Var71
Ec8
Var29
Var23
A2.1.1.2
Wld1
Orn9
A2.1.2
Var51
Var13
Var2
Var61
Var63
Ec97
Var18
A2.1.3_a
Ec36
Bl2
Bl6
A2.1.3_b
Var3
Var20
Var28
Ec65
Var4
Var15
Var1
Orn3
Ec9
Orn6
Orn4
Var32
Var27
Ec16
A2.2.1.1._a
Orn1
Hy2
Orn7
Orn13
A2.2.1.1._b
Var9
Var21
Var5
Var11
Var12
Var34
Orn12
A2.2.1.2
Orn10
Ec72
var39
Orn8
Orn2
Var6
Ec6
A2.2.2
Wld2
Var31
B1_a
Orn11
Bl4
B1_b
Var8
Var44
Hy8
B2
8
6
4
2
0
Branch Length

Figure S8

a)

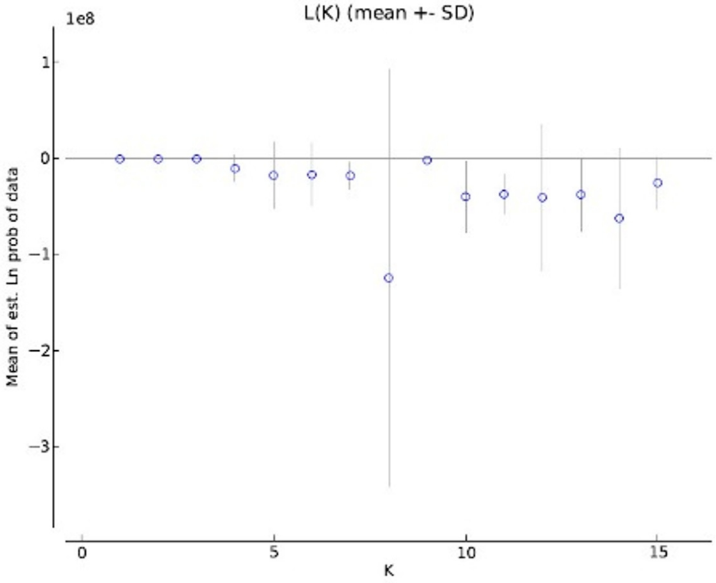
L(K) (mean +- SD)
1e8
1
0
−1
−2
−3
Mean of est. Ln prob of data
0
5
10
15
K

b)

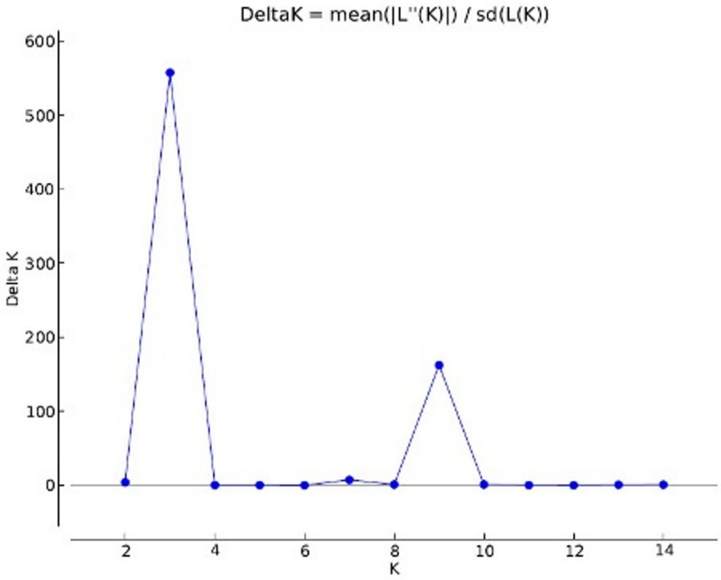
DeltaK = mean(|L''(K)|) / sd(L(K))
600
500
400
300
200
100
0
Delta K
2
4
6
8
10
12
14
K

Figure S9

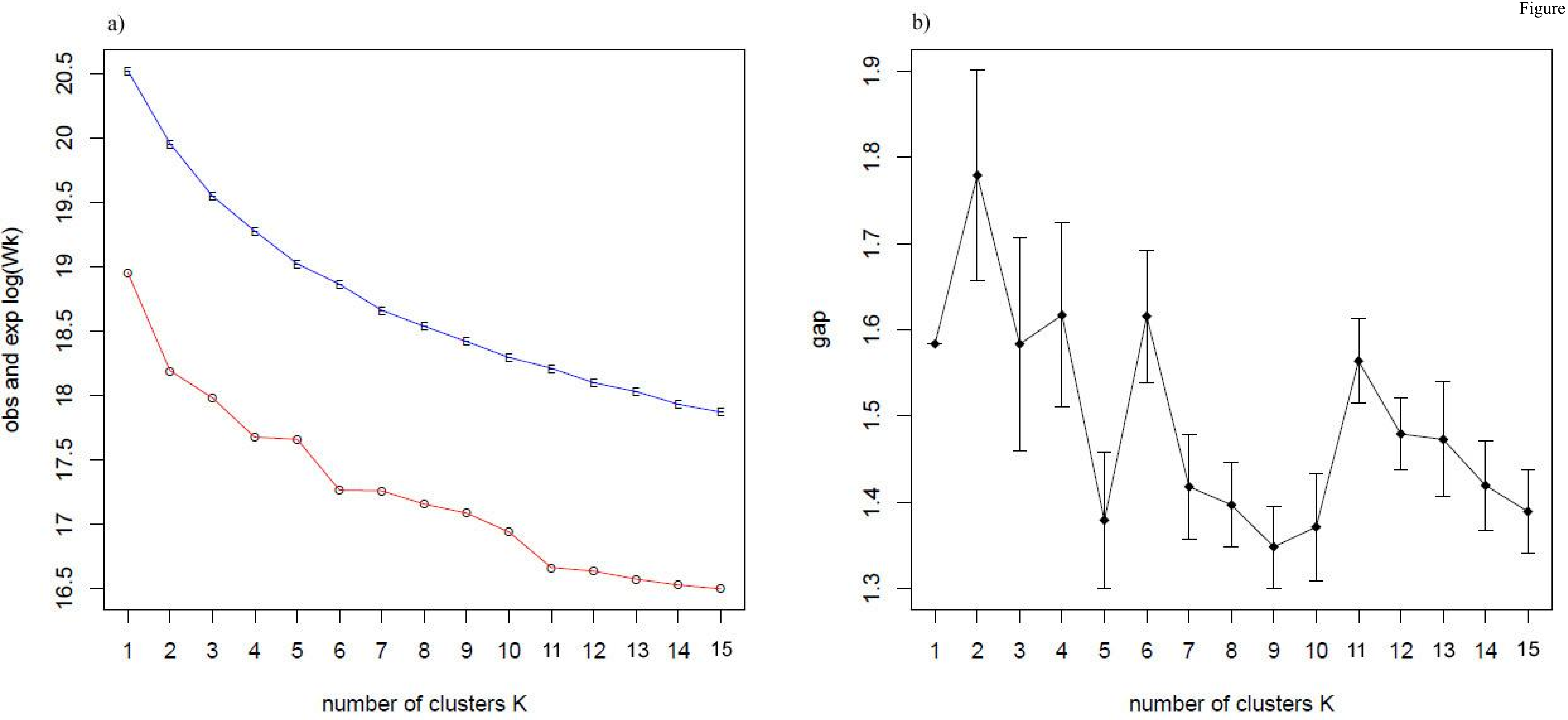
a)
obs and exp log(Wk)
number of clusters K
b)
gap
number of clusters K
